# Supplementary material for: Cortex folding by combined progenitor expansion and adhesion-controlled neuronal migration
Source: Nat Commun. 2025 Aug 28;16:8048. doi: 10.1038/s41467-025-62858-9 (PMC12394721; doi:10.1038/s41467-025-62858-9)
Supplement: Supplementary file 1 — Supplementary Information [file 41467_2025_62858_MOESM1_ESM.pdf]

Supplementary Information

Cortex Folding by Combined Progenitor Expansion and Adhesion-Controlled Neuronal Migration

Seung Hee Chun[1], Da Eun Yoon[2], D. Santiago Diaz Almeida[1], Mihail Ivilinov Todorov[3,4], Tobias Straub[7], Tobias Ruff[8], Wei Shao[9], Jianjun Yang[10], Gönül Seyit-Bremer[1], Yi-Ru Shen[1], Ali Ertürk[3,4,5,6], Daniel del Toro[11], Songhai Shi[10] and Rüdiger Klein[1*]

[1] Max Planck Institute of Biological Intelligence, Am Klopferspitz 18, 82152 Martinsried, Germany.

[2] Transgenic Core Facility, Max Plank Institute of Biochemistry, Am Klopferspitz 18, 82152 Martinsried, Germany.

[3] Institute for Stroke and Dementia Research (ISD), University Hospital, Ludwig-Maximilians-University Munich, 81377 Munich, Germany.

[4] Institute for Tissue Engineering and Regenerative Medicine (iTERM), Helmholtz Munich, 85764 Neuherberg, Germany.

[5] Munich Cluster for Systems Neurology (SyNergy), 80336 Munich, Germany.

[6] Koç Unive Koç University, School of Medicine, İstanbul, Turkeyrsity, School of Medicine, İstanbul, Turkey.

[7] Bioinformatics Core, Biomedical Center, Faculty of Medicine, Ludwig-Maximilians University (LMU), 82152 Martinsried, Germany.

[8] Laboratory of Biosensors and Bioelectronics, Institute for Biomedical Engineering, Eidgenössische Technische Hochschule (ETH) Zürich, 8092 Zürich, Switzerland.

[9] Biochemistry, Cell and Molecular Biology Allied Graduate Program, Weill Cornell Medical College, New York, NY, USA.

[10] New Cornerstone Science Laboratory, IDG/McGovern Institute for Brain Research, School of Life Sciences, Tsinghua University, 100084 Beijing, China.

[11] Department of Biomedical Sciences, Faculty of Medicine and Health Sciences, Institute of Neurosciences, IDIBAPS, University of Barcelona, 08036 Barcelona, Spain

[*] Corresponding author email: ruediger.klein@bi.mpg.de

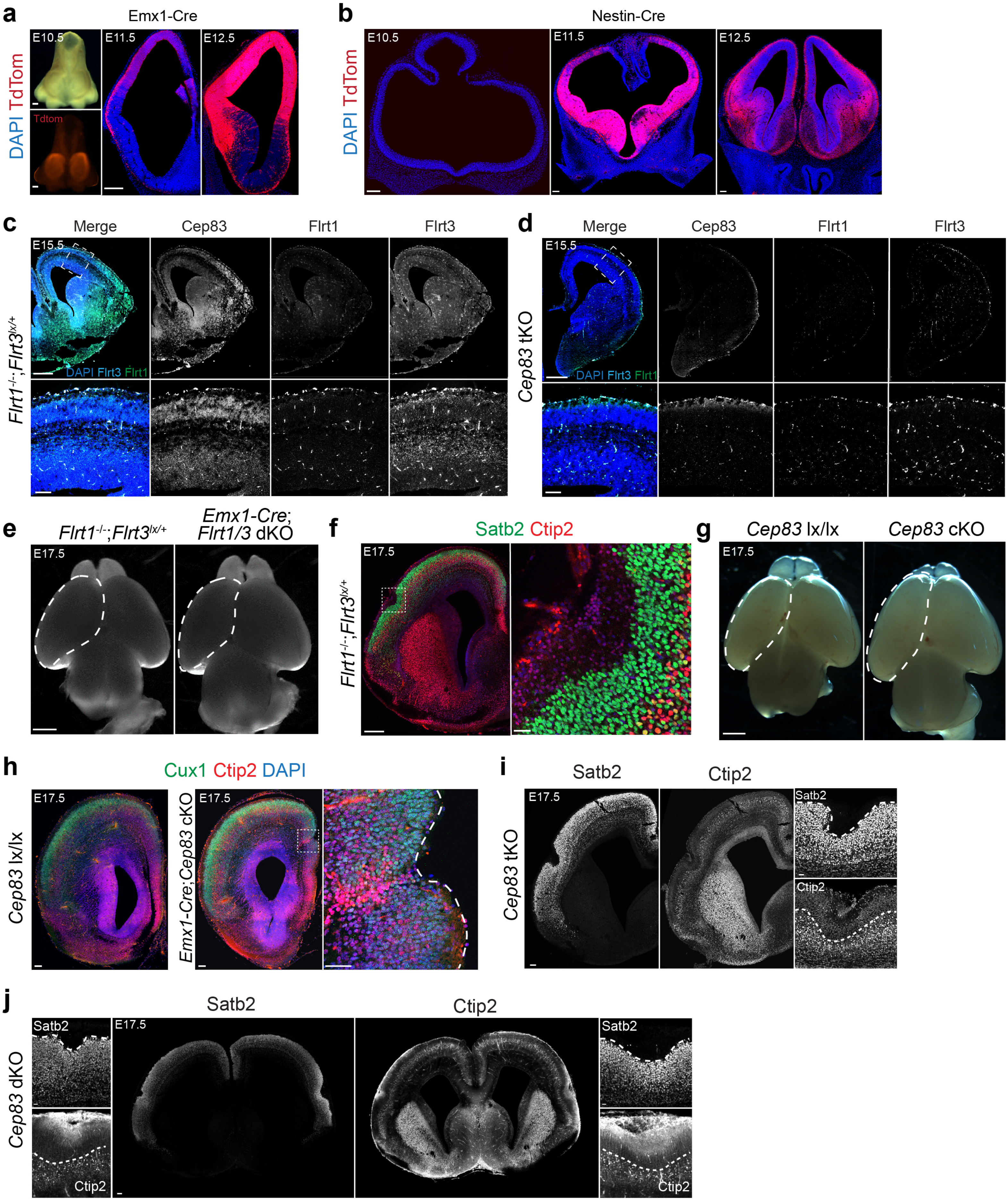

Supplementary Figure 1

Supplementary Fig. 1 Cep83 and Flrt1/Flrt3 loss enhances sulci-like cortex folding

a, Macroscopic image of E10.5 of Emx1-Cre embryo, head only (above). tdTomato expression of Emx1-Cre in embryo brain (below). The Cre-dependent reporter pCALNL-TdTom expression with DAPI in E11.5 and E12.5 of coronal view of cortex. Scale bars, 20 μm, 20 μm, 100 μm.

b, The Cre-dependent reporter pCALNL-TdTom expression tdTomato expression of Nestin-Cre with DAPI in E10.5, E11.5 and E12.5 of coronal view of cortex. Scale bars, 100 μm.

c, Double *in situ* hybridization (ISH) for Flrt1 and Flrt3 combined with Cep83 antibody staining in coronal sections of E15.5 cortex of *Flrt1*$^{-/-}$;*Flrt3*$^{lx/+}$. Area in dashed rectangle is shown with higher magnification on the below images. Scale bars, 500 μm, 100 μm.

d, Double ISH for Flrt1 and Flrt3 combined with Cep83 antibody staining in coronal sections of E15.5 cortex of *Cep83* tKO. Area in dashed rectangle is shown with higher magnification on the below images. Scale bars, 500 μm, 100 μm.

e, Representative whole-mount images of E17.5 *Flrt1* $^{-/-}$*Flrt3* $^{lx/+}$ and Emx1-Cre;*Flrt1/3* dKO brains. Dashed areas were measured to obtain quantification in Fig. 1a. Scale bar, 1mm.

f, E17.5 *Flrt1*$^{-/-}$;*Flrt3*$^{lx/+}$ brain section labeled with Satb2 (green), Ctip2 (red). A folding area in dashed rectangle is shown with higher magnification on the right. Scale bars, 1mm, 100 μm.

g, Representative whole-mount images of E17.5 *Cep83* lx/lx and *Cep83* cKO brains. Dashed areas were measured to obtain quantification in Fig. 1c. Scale bar, 1mm.

h, E17.5 *Cep83* lx/lx and Emx1-Cre;*Cep83* cKO brain sections labeled by Cux1 (green), Ctip2 (red), and DAPI (blue). Area in dashed rectangle is shown with higher magnification on the right. Scale bars, 100 μm.

i, Individual channels of whole section and higher magnification of sulcus area of Fig. 2c labeled with Satb2 (green), Ctip2 (red). Scale bars, 100 μm, 25 μm.

j, Individual channels of whole section and higher magnification of sulcus area of Fig. 2d labeled with Satb2 (green), Ctip2 (red). Scale bars, 50 μm, 100 μm, 50 μm.

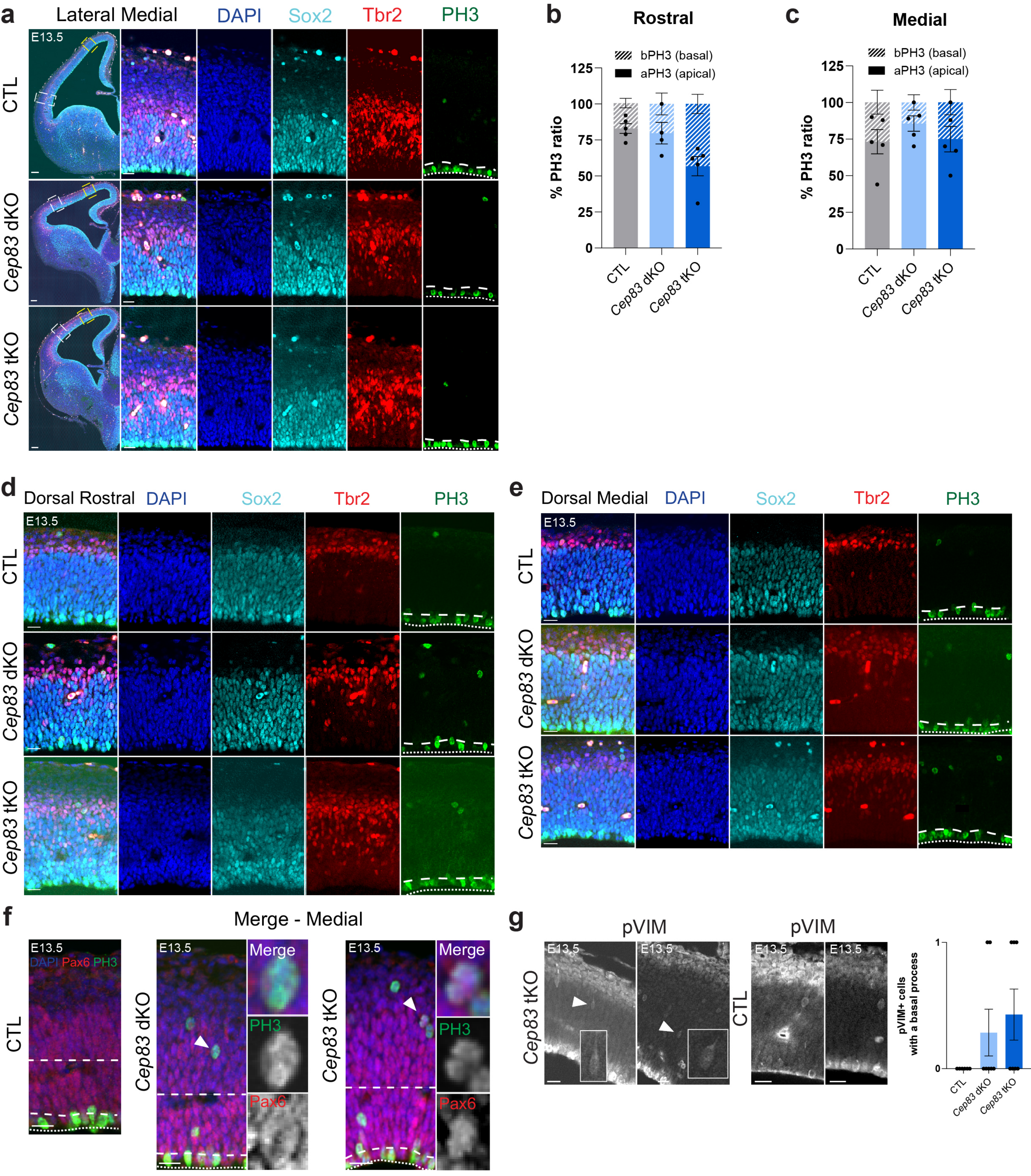

Supplementary Figure 2

Supplementary Fig. 2 Increased intermediate progenitors in *Cep83* tKO mice

a, E13.5 cortical sections of a medial region of CTL, *Cep83* dKO. and *Cep83* tKO embryos stained with DAPI (blue), Sox2 for early neuronal progenitors (cyan), Tbr2 for intermediate progenitors (red), and PH3 for mitotic cells (green). The apical side of the VZ is indicated with a dotted line, the basal side with a dashed line in PH3 stained images. Areas in white dashed rectangles in (**a**, from the lateral side) are shown with higher magnification on the right. Areas in yellow dashed rectangles are from the dorsal side. Scale bars,100 µm, 50 µm.

b, Proportion of apical/basal mitotic cells (PH3) in lateral area of rostral region (CTL n = 5 brains, *Cep83* dKO n = 5 brains, *Cep83* tKO, n = 5 brains from 3litters). Data are shown as mean ± SEM; aPH: CTL vs *Cep83* dKO, p = 0.486, CTL vs *Cep83* tKO, p = 0.093, *Cep83* dKO vs *Cep83* tKO, p = 0.516, bPH: CTL vs *Cep83* dKO, p = 0.515, CTL vs *Cep83* tKO, p = 0.101, *Cep83* dKO vs *Cep83* tKO, p = 0.515. one-way ANOVA with Tukey's post hoc analysis.

c, Proportion of apical/basal mitotic cells (PH3) in lateral area of medial region (CTL n = 5 brains, *Cep83* dKO n = 5 brains, *Cep83* tKO, n = 5 brains from 3litters). Data are shown as mean ± SEM; aPH: CTL vs *Cep83* dKO, p = 0.497, CTL vs *Cep83* tKO, p = 0.985, *Cep83* dKO vs *Cep83* tKO, p = 0.595, bPH: CTL vs *Cep83* dKO, p = 0.481, CTL vs *Cep83* tKO, p = 0.984, *Cep83* dKO vs *Cep83* tKO, p = 0.579. one-way ANOVA with Tukey's post hoc analysis.

d, High magnification images of E13.5 dorsal areas of rostral cortices of CTL, *Cep83* dKO. and *Cep83* tKO embryos stained as in Fig. 2a. Scale bars, 20 µm.

e, Similar experiment as described in **d** except for dorsal areas of medial cortices shown in **a**. Scale bars, 20 µm.

f, High magnification images of E13.5 medial cortical sections of CTL, *Cep83* dKO. and *Cep83* tKO embryos were stained with DAPI (blue), Pax6 for RG cells (red), PH3 for mitotic cells (green). The apical side of the VZ is indicated with a dotted line, the basal side with a dashed line in Pax6/PH3 images. The Pax6/PH3 co-immunopositive cells located more than 60µm (approximately three nuclei) above the apical surface, outside the VZ Cells marked by arrowheads are shown in higher magnification in the right. Scale bars, 20 µm.

g, Quantification of pVIM+ cells with a basal process (marked with an arrowhead). CTL n = 6 brains, *Cep83* dKO n = 7 brains, *Cep83* tKO, n = 7 brains from 4 litters). Data are shown as mean ± SEM. Higher magnification images depict the basal process of pVIM+ cells in *Cep83* tKO embryos. Graph on the right shows total number of pVIM cells with a basal process in each genotype. Scale bars, 20 µm.

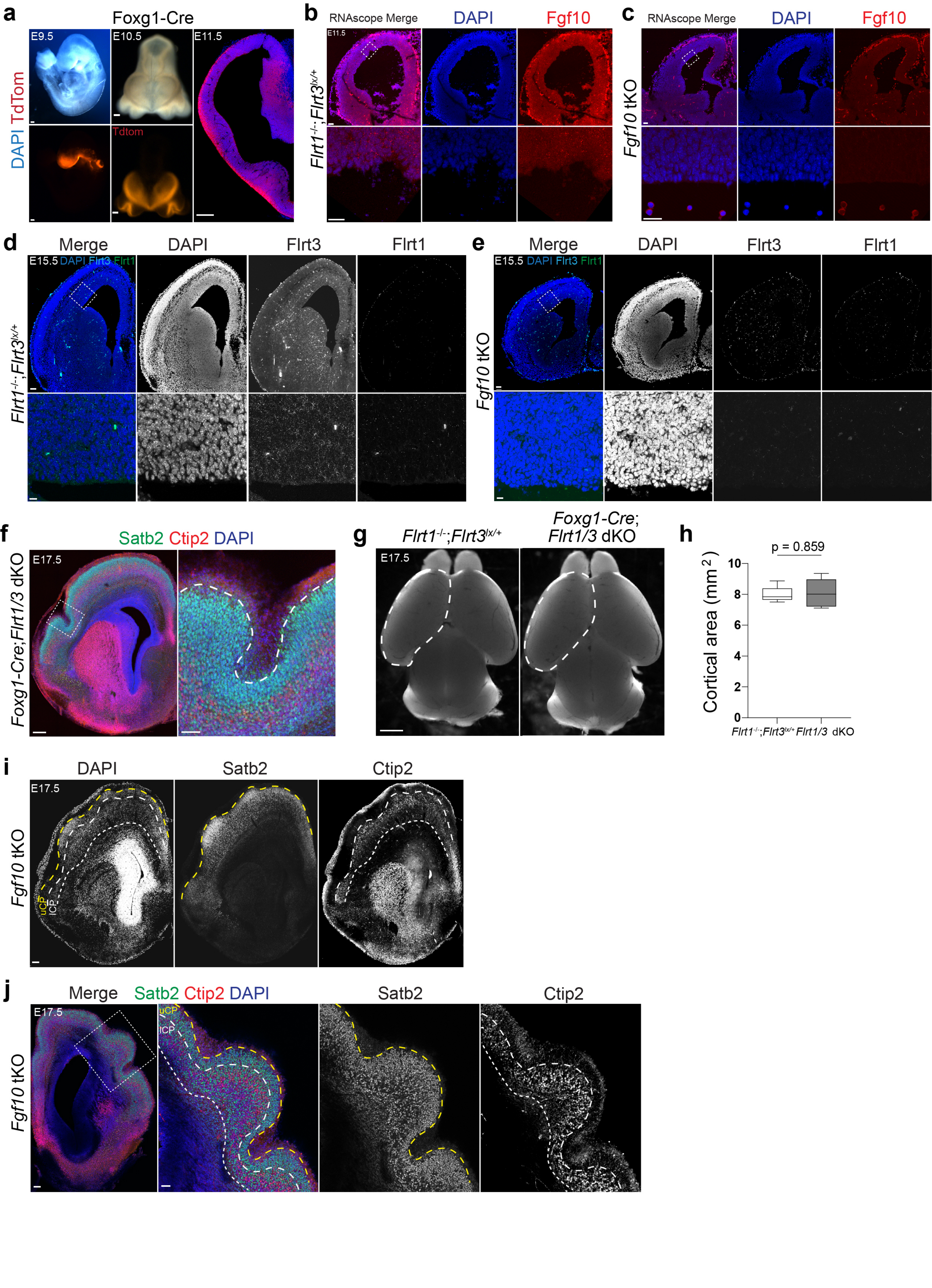

Supplementary Figure 3

Supplementary Fig. 3 Fgf10 and Flrt1/Flrt3 loss enhances gyri-like cortex folding

a, Macroscopic image of E9.5, E10.5 and E11.5 of Foxg1-Cre embryo. E9.5 whole embryo and E10.5 head only (above) is shown with the Cre-dependent reporter pCALNL-TdTom expression of Foxg1-Cre in embryo brain (below) and with DAPI in E11.5 of coronal view of cortex. Scale bars, 100 µm, 20 µm, 100 µm, 20 µm, 100 µm.

b, ISH for Fgf10 in coronal sections of E11.5 cortex of *Flrt1*$^{-/-}$;*Flrt3*$^{lx/+}$. Area in dashed rectangle is shown in higher magnification of the VZ. Scale bars, 50 µm, 25 µm.

c, ISH for Fgf10 in coronal sections of E11.5 cortex of *Fgf10* tKO. Area in dashed rectangle is shown in higher magnification of the VZ. Scale bars, 50 µm, 25 µm.

d, Double ISH for Flrt1 and Flrt3 with DAPI in coronal sections of E15.5 cortex of *Flrt1*$^{-/-}$;*Flrt3*$^{lx/+}$. Area in dashed rectangle is shown with higher magnification on the below images. Scale bars, 100 µm, 10 µm.

e, Double ISH for Flrt1 and Flrt3 with DAPI in coronal sections of E15.5 cortex of *Fgf10* tKO. Scale bars, Area in dashed rectangle is shown with higher magnification on the below images. Scale bars, 100 µm, 10 µm.

f, Representative E17.5 *Foxg1-Cre*;*Flrt1/3* dKO brain section labeled with Satb2 (green), Ctip2 (red), and DAPI (blue). Area in dashed rectangle is shown with higher magnification on the right. Dashed line indicate a sulcus. Scale bars, 200 µm, 50 µm.

g, Representative whole-mount images of E17.5 *Flrt1* $^{-/-}$*Flrt3* $^{lx/+}$ and *Foxg1-Cre*;*Flrt1/3* dKO brains. Dashed area were measured to obtain quantifications. Scale bar, 1mm.

h, Quantifications of the cortical areas shown in **g**. *Flrt1* $^{-/-}$*Flrt3* $^{lx/+}$, n = 10 brains; Foxg1-Cre;*Flrt1/3* dKO, n = 11 brains from 6 litters, two-tailed t-test with Welch correction.

i, E17.5 *Fgf10* tKO brain section shown in Fig. 5b immunostained with DAPI (grey), Satb2 (grey) and Ctip2 (grey). DAPI channel; uCP and lCP are delineated by yellow and white dashed lines, respectively. To distinguish these layers sections were immunostained with Satb2 and Ctip2. Scale bar, 100 µm.

j, E17.5 *Fgf10* tKO brain section stained with Satb2, Ctip2, and DAPI (merge). Single gyrus outlined by dotted square. Higher magnifications on the right. Merge channel; uCP and lCP are delineated by yellow and white dashed lines, respectively. To distinguish these layers sections were immunostained with Satb2 and Ctip2. Scale bars, 100 µm, 50 µm.

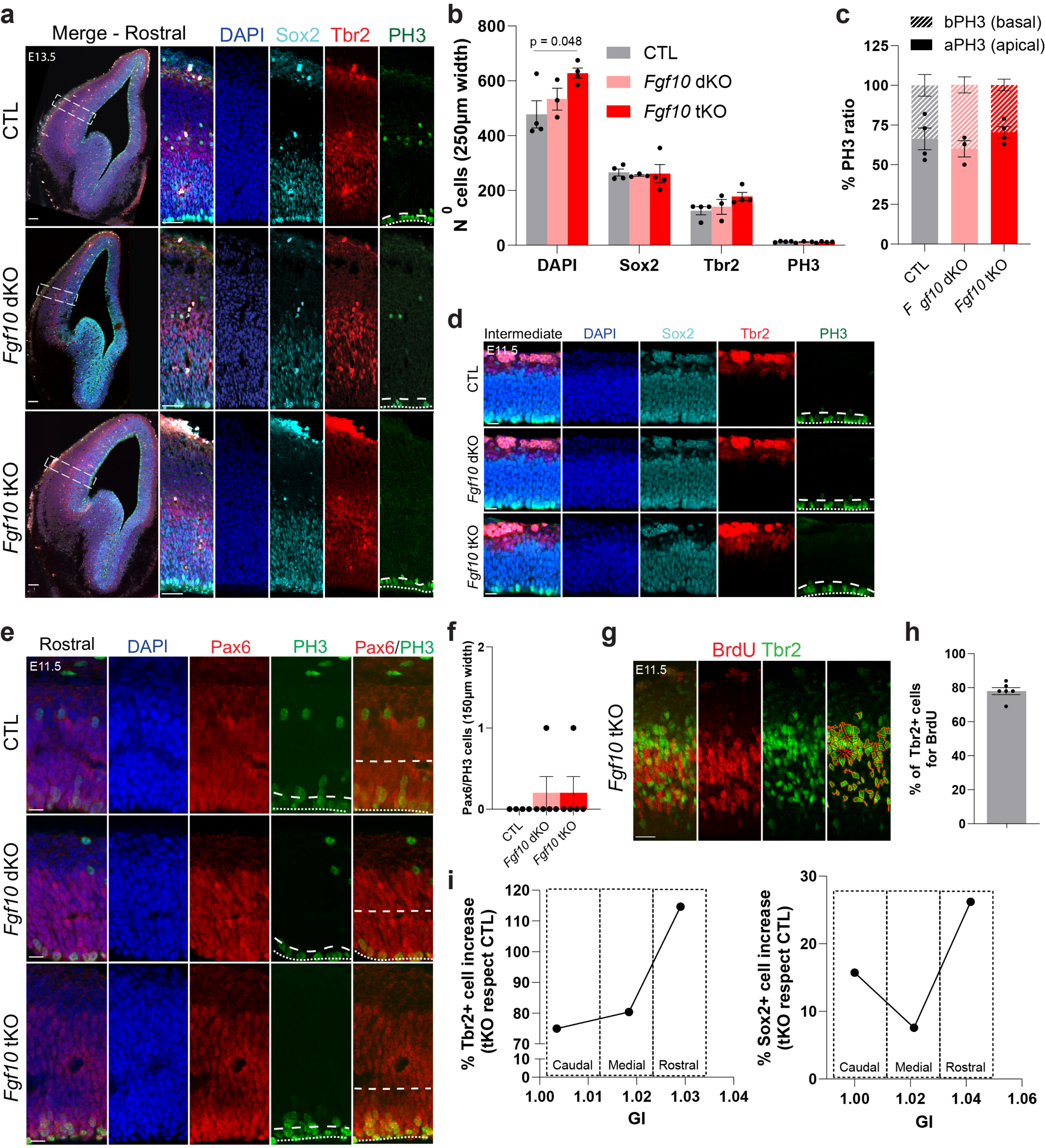

Supplementary Figure 4

Supplementary Fig. 4 Progenitor expansion in *Fgf10* tKO *and Fgf10* dKO embryos at E11.5/E13.5

a, E13.5 rostral cortices of CTL, *Fgf10* dKO and *Fgf10* tKO embryos were stained with DAPI (blue), apical progenitors Sox2 (cyan), intermediate progenitors Tbr2 (red), and mitotic cells PH3 (green). Apical and basal sides of the VZ are indicated with dotty and dashed lines in PH3 stained images, respectively. Areas in dashed rectangles in **a** are shown with higher magnification on the right. Scale bars,100 µm, 50 µm.

b, Quantification of the data shown in **a**. CTL, n = 4 brains, *Fgf10* dKO n = 3 brains, *Fgf10* tKO n = 4 brains from 3 litters. Data are shown as mean ± SEM; CTL vs *Fgf10* tKO. one-way ANOVA with Tukey's post hoc analysis.

c, Proportion of apical/basal mitotic cells (PH3) in rostral region CTL, n = 4, *Fgf10* dKO n = 3, *Fgf10* tKO n = 4 from 3 liters. Data are shown as mean ± SEM; no significant changes between group, one-way ANOVA with Tukey's post hoc analysis.

d, E11.5 cortices from intermediate regions of CTL, *Fgf10* dKO and *Fgf10* tKO embryos stained as in panel **a**. Scale bars, 20 µm.

e, E11.5 rostral cortices of CTL, *Fgf10* dKO. and *Fgf10* tKO embryos stained with DAPI (blue), Pax6 for RG cells (red), PH3 for mitotic cells (green). The apical side of the VZ is indicated with a dotted line, the basal side with a dashed line in Pax6/PH3 images. The Pax6/PH3 co-immunopositive cells located more than 60µm (approximately three nuclei) above the apical surface, outside the VZ Cells. Scale bars, 50 µm.

f, Quantifications of Pax6/PH3 co-positive cell densities in the rostral regions shown in **e**. CTL, n = 4 brains, *Fgf10* dKO n = 5 brains, *Fgf10* tKO, n = 5 brains from 3 litters). Data are shown as mean ± SEM; no significant changes between groups, one-way ANOVA with Tukey's post hoc analysis.

g, BrdU injection was performed at E11.5 and collected after 30 min in *Fgf10* tKO embryos. BrdU expression was confirmed by immunostaining rostral sections against BrdU (red) together with the IP marker Tbr2 (green). Tbr2+/BrdU+ cells are outlined in red in the Tbr2 stained image. Scale bar, 25 µm.

h, Percentage of Tbr2+ cells among BrdU+ cells. Quantification of the data shown in **g**. n = 6 sections of 4 brains from 3 litters.

i, Correlation between the degree of cortical folding, measured as the gyrification index (GI), and the % increase in progenitor cells (Tbr2 for *Cep83* dKO/tKO and Sox2 for *Fgf10* dKO/tKO) respect to controls.

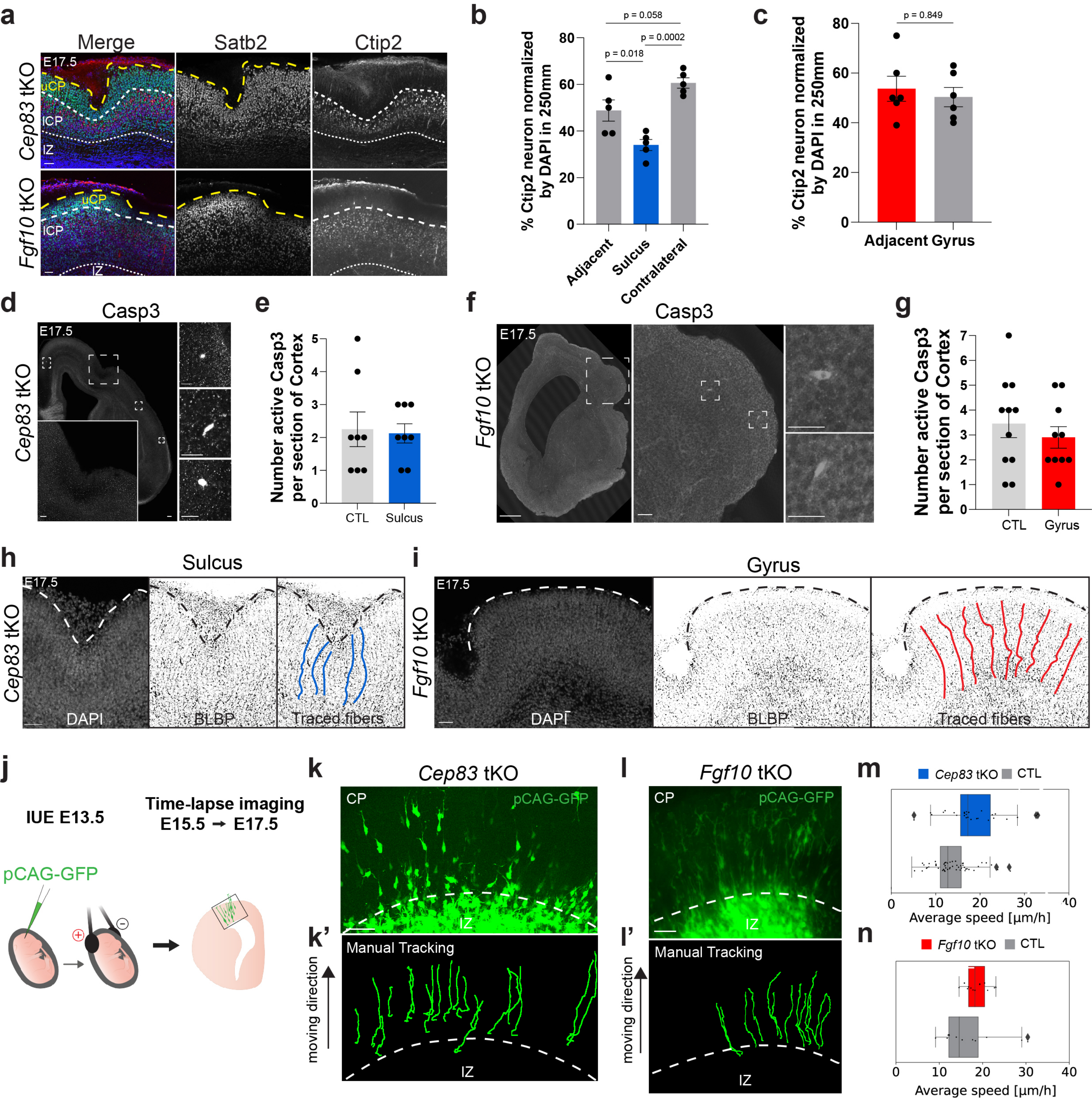

Supplementary Figure 5

Supplementary Fig. 5 Higher cell density in gyri of *Fgf10* tKO mice

a, E17.5 *Cep83* tKO and *Fgf10* tKO cortical sections labeled with Satb2 (green), Ctip2 (red), and DAPI (blue). Images on the right show the CP upper layer marker, Satb2 (grey), and lower layer marker Ctip2 (grey). Boundaries of upper (yellow) and lower (white) layers are indicated. uCP, upper cortical plate layer, lCP, lower cortical plate layer, IZ, intermediate zone. Scale bars, 50 µm.

b, Quantification of the data shown in Fig. 8a. Ctip2+ cells were counted in the sulcus, adjacent and contralateral regions of the cortex (n = 6 embryos from 5 litter). Data are shown as mean ± SEM. one-way ANOVA with Tukey's post hoc analysis.

c, Quantification of the data shown in Fig. 8b. Satb2+ cells were counted in gyri and adjacent region of the cortex. (n = 6 embryos from 5 litter). Data are shown as mean ± SEM. Two-tailed t-test with Welch correction.

d, E17.5 *Cep83* tKO brain section labeled with Casp3, an apoptosis marker. Dashed rectangle areas are shown with higher magnification on the left (sulcus area) and right (Casp3 positive cells). Scale bars, 300 µm, 50 µm, 25 µm, 25 µm, 25 µm.

e, Quantification of the data shown in **d**. CTL, n = 8 sections from 4 embryos from 3 litters, Sulcus n = 8 sections from 6 embryos from 3 litters.

f, E17.5 *Fgf10* tKO brain section labeled with Casp3. Dashed rectangle areas are shown on the right (Casp3 positive cells) with higher magnification. Scale bars, 300 µm, 50 µm, 25 µm, 25 µm.

g, Quantification of the data shown in **f**. CTL, n = 11 sections from 6 embryos from 5 litters. Gyrus n = 10 sections from 8 embryos from 5 litters.

h, E17.5 *Cep83* tKO brain section labeled with DAPI and BLBP, a RG fiber marker. Dashed line indicates sulcus. Single traced RG fibers in the sulcus region are colored in blue. Scale bar, 50 µm.

i, E17.5 *Fgf10* tKO brain section labeled with DAPI and BLBP. Dashed line indicates gyrus. Single traced RG fibers in the gyrus region are colored in red. Scale bar, 50 µm.

j, Schematic diagram of in utero electroporation (IUE) performed at E13.5 of embryo. The time-lapse imaging starts at E15.5 with ex vivo sliced section labeled by pCAG-Ires-GFP by IUE over 48 hours.

k, Migrating GFP l—abelled neurons by IUE were tracked in the CP in ex vivo sliced section of *Cep83* tKO. **k'**, Migration paths of manually tracked neurons within CP for speed analysis. Scale bar, 50 µm.

l, Migrating GFP labelled neurons by IUE was tracked in the CP in ex vivo sliced section of *Fgf10* tKO. **l'**, Progression lines of tracked migrating neurons within CP for speed analysis. Scale bar, 50 µm.

m, Quantification of the tracked neurons in **k'**. The average speed of tracked neurons is represented as a box plot, with median (centre line), 50% interquartile range (box) and whiskers extending 1.5 times the interquartile range; Average speed values of individual neurons are represented as a scatterplot, *Cep83* tKO, n = 2 embryos, CTL (*Flrt1*$^{-/-}$;*Flrt3*$^{lx/+}$), n = 3 embyos from 3 litters.

n, Quantification of tracked neurons in **l'**. The average speed of tracked neurons is represented as a box plot, with median (centre line), 50% interquartile range (box) and whiskers extending 1.5 times the interquartile range; Average speed values of individual neurons are represented as a scatterplot; *Fgf10* tKO, n =1 embryo, CTL, n = 1 embryo.

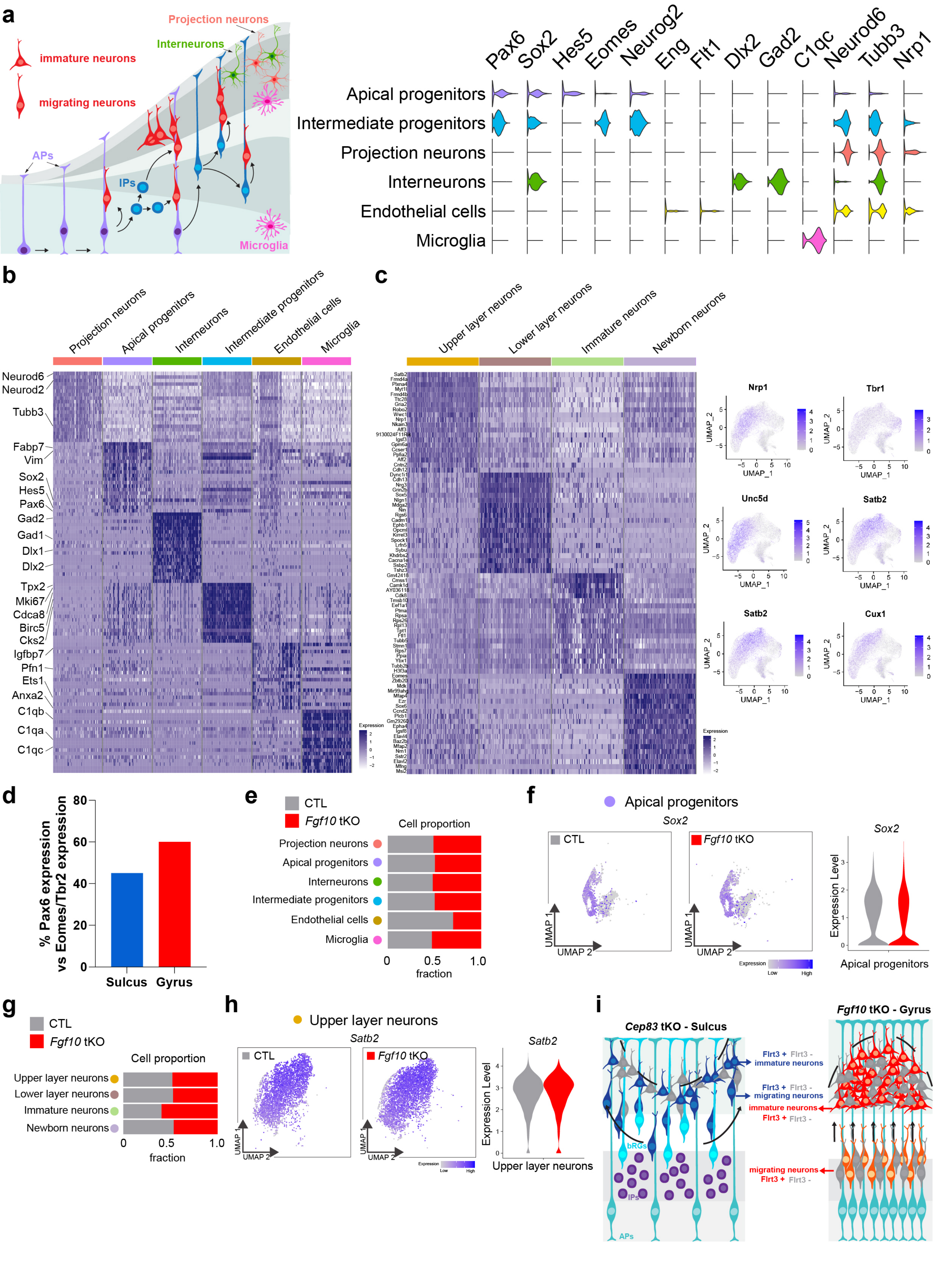

Supplementary Figure 6

Supplementary Fig.6 Modeling cortex folding in *Cep83* tKO and *Fgf10* tKO mice

a, Expression of marker genes per cell type. Cell types are grouped on the basis of their identity and shared maker genes.

b, Gene signatures for all cell types identified in the combined all genotypes. Top 30 differentially expressed genes for each cell type are presented. Cells were down-sampled to a maximum of 100 cells per cell type.

c, Gene signatures for all cell types identified in the combined all genotypes. Top 30 differentially expressed genes for each cell type are presented. Cells were down-sampled to a maximum of 100 cells per cell type Expression of canonical marker genes for selected cell types in the UMAP visualization of the combined all genotypes.

d, Expression proportion between Pax6 and Eomes/Tbr2 in the germinal zone of both sulci and gyri using RNA profiling data from ferret samples, published in Del-Valle-Anton et al, 2024. (GSE234305).

e, Proportion of each cell type by genotype in CTL and *Fgf10* tKO mice.

f, Expression of the apical progenitor marker gene Sox2 in apical progenitor cell type for CTL (*Flrt1*$^{-/-}$;*Flrt3*$^{lx/+}$) and *Fgf10* tKO embryos. (UMAP and violin plot).

g, Proportion of sub cell type by genotype in CTL and *Fgf10* tKO mice.

h, Expression of CP layer marker gene, Satb2, in upper layer neurons for CTL) and *Fgf10* tKO embryos. (UMAP and violin plots).

i, Graphic summary of how folds develop in *Cep83* tKO (Sulcus) and *Fgf10* tKO (Gyrus) mice.
