# Supplementary material for: Cortex folding by combined progenitor expansion and adhesion-controlled neuronal migration
Source: Nat Commun. 2025 Aug 28;16:8048. doi: 10.1038/s41467-025-62858-9 (PMC12394721; doi:10.1038/s41467-025-62858-9)
Supplement: Supplementary file 2 — Description of Additional Supplementary Files [file 41467_2025_62858_MOESM2_ESM.pdf]

Description of Additional Supplementary Files

File Name: Supplementary Video 1

Description: 3D light sheet imaging of a whole brain stained with Satb2(green) and Ctip2(red) of E17.5 Cep83 tKO.

File Name: Supplementary Video 2

Description: Higher magnification of 3D imaging of half hemisphere shown in MovieS1

File Name: Supplementary Dataset 1

Description: : Primer list of genotyping for mutant : Emx1-Cre;Cep83lx/lx;Flrt1-/- ; Flrt3lx/lacZ mice and Foxg1-Cre;Fgf10lx/lx;Flrt1-/- ; Flrt3lx/lacZ mice. Each mouse was genotyped by PCR to differentiate KO (-/-) or heterozygous (lox/+) and LacZ.
