# Supplementary material for: Cortex folding by combined progenitor expansion and adhesion-controlled neuronal migration
Source: Nat Commun. 2025 Aug 28;16:8048. doi: 10.1038/s41467-025-62858-9 (PMC12394721; doi:10.1038/s41467-025-62858-9)
Supplement: Supplementary file 6 — Reporting Summary [file 41467_2025_62858_MOESM6_ESM.pdf]

nature portfolio

Corresponding author(s): Dr.Rüdiger Klein

Last updated by author(s): Jun 10, 2025

Reporting Summary

Nature Portfolio wishes to improve the reproducibility of the work that we publish. This form provides structure for consistency and transparency in reporting. For further information on Nature Portfolio policies, see our Editorial Policies and the Editorial Policy Checklist.

Statistics

For all statistical analyses, confirm that the following items are present in the figure legend, table legend, main text, or Methods section.

n/a	Confirmed	
☐	☒	The exact sample size (*n*) for each experimental group/condition, given as a discrete number and unit of measurement
☐	☒	A statement on whether measurements were taken from distinct samples or whether the same sample was measured repeatedly
☐	☒	The statistical test(s) used AND whether they are one- or two-sided<br>*Only common tests should be described solely by name; describe more complex techniques in the Methods section.*
☒	☐	A description of all covariates tested
☐	☒	A description of any assumptions or corrections, such as tests of normality and adjustment for multiple comparisons
☐	☒	A full description of the statistical parameters including central tendency (e.g. means) or other basic estimates (e.g. regression coefficient) AND variation (e.g. standard deviation) or associated estimates of uncertainty (e.g. confidence intervals)
☐	☒	For null hypothesis testing, the test statistic (e.g. *F*, *t*, *r*) with confidence intervals, effect sizes, degrees of freedom and *P* value noted<br>*Give P values as exact values whenever suitable.*
☒	☐	For Bayesian analysis, information on the choice of priors and Markov chain Monte Carlo settings
☒	☐	For hierarchical and complex designs, identification of the appropriate level for tests and full reporting of outcomes
☒	☐	Estimates of effect sizes (e.g. Cohen's *d*, Pearson's *r*), indicating how they were calculated

Our web collection on statistics for biologists contains articles on many of the points above.

Software and code

Policy information about availability of computer code

Data collection	ImageJ (Fiji), version 2.0.0-rc-69/1.53t, Rstudio, LAS software, Python, version 3.9.12, CellProfiler, version 4.2.5, Imaris, version 9.5.1,Seurat, version 4.3.0,Cellranger, version 7.0.1,GraphPad Prism, version 9/10,MATLAB, version R2015a,MATLAB particles, version 2.1
Data analysis	ImageJ (Fiji), version 2.0.0-rc-69/1.53t, Rstudio, LAS software, Python, version 3.9.12, CellProfiler, version 4.2.5, Imaris, version 9.5.1,Seurat, version 4.3.0,Cellranger, version 7.0.1,GraphPad Prism, version 9/10,MATLAB, version R2015a,MATLAB particles, version 2.1

For manuscripts utilizing custom algorithms or software that are central to the research but not yet described in published literature, software must be made available to editors and reviewers. We strongly encourage code deposition in a community repository (e.g. GitHub). See the Nature Portfolio guidelines for submitting code & software for further information.

Data

Policy information about availability of data

All manuscripts must include a data availability statement. This statement should provide the following information, where applicable:

- Accession codes, unique identifiers, or web links for publicly available datasets
- A description of any restrictions on data availability
- For clinical datasets or third party data, please ensure that the statement adheres to our policy

scRNA datasets used in this study are publicly available at the NCBI Gene Expression Omnibus (GEO) database (www.ncbi.nlm.nih.gov/geo) with accession numbers GSE267391. Single-cell RNA-seq data for gyri and sulci from ferret samples were obtained from the published NCBI Gene Expression Omnibus (GEO) database with

accession numbers GSE234305. The Time-lapse experiments, Single cell track analysis data generated in this study are provided in this study are available https://doi.org/10.5281/zenodo.15522270. The MATLAB base code for Computational simulations data generated in this study are available https://doi.org/10.5281/zenodo.15583689.

Research involving human participants, their data, or biological material

Policy information about studies with human participants or human data. See also policy information about sex, gender (identity/presentation), and sexual orientation and race, ethnicity and racism.

Reporting on sex and gender	No human participants or human data were used in study.
Reporting on race, ethnicity, or other socially relevant groupings	No human participants or human data were used in study
Population characteristics	No human participants or human data were used in study
Recruitment	No human participants or human data were used in study
Ethics oversight	No human participants or human data were used in study

Note that full information on the approval of the study protocol must also be provided in the manuscript.

Field-specific reporting

Please select the one below that is the best fit for your research. If you are not sure, read the appropriate sections before making your selection.

☒ Life sciences ☐ Behavioural & social sciences ☐ Ecological, evolutionary & environmental sciences

For a reference copy of the document with all sections, see nature.com/documents/nr-reporting-summary-flat.pdf

Life sciences study design

All studies must disclose on these points even when the disclosure is negative.

Sample size	No sample size calculations were performed. Sample size was determined ti be adequate based on the magnitude and consistency of measurable difference between groups.
Data exclusions	Data were only excluded for failed experiments
Replication	The number of times each experiment was repeated independently with similar results were provided in the figure ledends
Randomization	No randomization of samples was performed
Blinding	Investigator were not blinded to mouse genotypes during experiments but for analysis was done blindly.

Reporting for specific materials, systems and methods

We require information from authors about some types of materials, experimental systems and methods used in many studies. Here, indicate whether each material, system or method listed is relevant to your study. If you are not sure if a list item applies to your research, read the appropriate section before selecting a response.

Materials & experimental systems

n/a	Involved in the study	
☐	☒	Antibodies
☒	☐	Eukaryotic cell lines
☒	☐	Palaeontology and archaeology
☐	☒	Animals and other organisms
☒	☐	Clinical data
☒	☐	Dual use research of concern
☒	☐	Plants

Methods

n/a	Involved in the study	
☒	☐	ChIP-seq
☒	☐	Flow cytometry
☒	☐	MRI-based neuroimaging

Antibodies

Antibodies used	Provided in Method section. Rabbit anti SATB2 1:500 (Abcam, Cat # AB34735; RRID:AB_2301417), Rat anti Ctip2 1:500 (Abcam, Cat # AB18465; RRID:AB_2064130), Goat anti Sox2 1:1000 (R&D Systems; Cat # AF2018; RRID:AB_355110) Rat anti Histone H3 1:500 (Abcam, Cat # AB10543; RRID:AB_2295065), Rabbit anti-Tbr1 1:500 (Abcam, Cat#AB31940; RRID:AB_2200219), Rabbit anti Tbr2/ Eomes 1:200 (Abcam, Cat # AB183991; RRID:AB_2721040), Rat anti BrdU 1:100 (Abcam, Cat # AB6326; RRID:AB_305426) Rabbit anti-Cux1 1:500 (Santa Cruz, Cat # SC-13024; RRID:AB_2261231), Rabbit anti-BLBP 1:300 (Millipore, Cat#ABN14; RRID:AB_10000325), Rabbit anti-Pax6 1:300 (BioLegend, Cat#901301; RRID:AB_2565003), rabbit anti-cleaved Caspase 3 1:300 (Cell Signaling Technology, Inc, Cat#9661; RRID:AB_2341188), mouse anti-Pvim 1:500 (Abcam, Cat# AB22651; RRID:AB_447222), Secondary antibodies were diluted at 1:500, Alexa Fluor 647 AffiniPure Donkey Anti- Goat(Jackson Immuno Research, Cat # 705-605-147; RRID:AB_2340437), Cy3 AffiniPure Donkey Anti - Rabbit(Jackson Immuno Research, Cat # 711-165-152; RRID:AB_2307443), Alex Fluor 488 AffiniPure Donkey Anti - Rat(Jackson Immuno Research, Cat # 712-545-153; RRID:AB_2340684), Cy3 AffiniPure Donkey Anti - Mouse(Jackson Immuno Research, Cat # 715-165-150; RRID:AB_2340813), Cy3 AffiniPure Donkey Anti - Rat (Jackson Immuno Research, Cat # 712-165-153; RRID: AB_2340667), Alex Fluor 488 AffiniPure Donkey Anti - Rabbit (Jackson Immuno Research, Cat # 711-545-152; RRID:AB_2313584)
Validation	All antibodies are commercially available.

Animals and other research organisms

Policy information about studies involving animals; ARRIVE guidelines recommended for reporting animal research, and Sex and Gender in Research

Laboratory animals	FLRT1 null (Yamagishi et al., 2011), FLRT3 lox (Yamagishi et al., 2011), FLRT3 lacZ (Egea et al., 2008), Cep83 lox (Shao et al., 2020), Fgf10 lox B6;129-Fgf10<tm1.2Sms>/J (The Jackson Laboratory; RRID:IMSR_JAX:023729), Emx1-Cre B6.Cg-Emx1tm1(cre)Kr (Gorski et al., 2002; RRID:IMSR_JAX:005628), Foxg1-IRES-Cre B6.129T(SJL)-Foxg1tm1.1(cre)Ddmo/J (The Jackson Laboratory, RRID:IMSR_JAX:029690), B6.Cg-Gt(ROSA)26Sortm9(CAG-tdTomato)Hze/J (The Jackson Laboratory, RRID:IMSR_JAX:00790)
Wild animals	No wild animal was used
Reporting on sex	Both male and females were used
Field-collected samples	No field-collected sample was used
Ethics oversight	All animal experiments were approved by the Government of Upper Bavaria and carried out in accordance with German guidelines for animal welfare. All mice (C57BL/6 and 129/SvJ mixed background) were housed with 12:12h light/dark cycle and food/water available ad libitum in the facilities of the Max Planck Institute of Biological Intelligence. License number ROB 55.2-2532.Vet_02-20-2 until 16.10.2025

Note that full information on the approval of the study protocol must also be provided in the manuscript.

Plants

Seed stocks	No plants were used in study
Novel plant genotypes	No plants were used in study
Authentication	No plants were used in study
